# Efficacy and Safety of the Pneumococcal Conjugate-13 Valent Vaccine in Adults

**DOI:** 10.14336/AD.2018.0512

**Published:** 2019-04-01

**Authors:** Fawziah Marra, Nirma Khatri Vadlamudi

**Affiliations:** Faculty of Pharmaceutical Sciences, University of British Columbia, Vancouver, Canada

**Keywords:** Immunization, S. pneumonia or pneumococcus, Streptococcus pneumoniae, pneumococcal conjugate vaccines, PCV13, older adults, effectiveness, efficacy

## Abstract

Invasive pneumococcal disease and pneumococcal pneumonia cause substantial morbidity and mortality in the elderly. This review focuses on the immunogenicity, safety, efficacy and effectiveness data on the use of the 13-valent conjugate pneumococcal vaccine (PCV13) in adults. A MEDLINE literature search was performed from January 1946 to December 2017. Additional references were identified from a review of literature citations. All English-language randomized trials, observational studies and meta-analyses assessing the immunogenicity, efficacy, effectiveness and safety of PCV13 in adults were evaluated. Six randomized controlled studies evaluated immunogenicity and safety of PCV13 in adults and showed that the conjugated vaccine elicited a greater immune response to the majority of the 13 serotypes compared to the 23-valent polysaccharide pneumococcal vaccine (PPV23). Administering PCV13 prior to PPV23 elicits greater immune responses and multiple doses of PCV13 demonstrated modest advantage. PCV13 titers declined after a year but remained above baseline. A randomized clinical trial (CAPiTA) showed that PCV13 was effective in preventing community-acquired pneumonia (CAP) and vaccine-type invasive pneumococcal disease, but not any cause pneumonia. Safety data shows PCV13 elicits minor local reactions, such as pain at the injection site. Major side effects that were commonly reported included muscle fatigue and headache. Both local and systemic adverse events were comparable to PPV23. While PCV13 has a well-established immunogenicity and safety profile in adults, there is sparse data on sequential or multiple dosing, efficacy and effectiveness in adults. As there are few countries who have adopted PCV13 for routine adult immunization, there is a need to evaluate the effectiveness of PCV13 in a real-world setting.

The World Health Organization (WHO) estimates pneumococcal disease affects 14 million people and causes approximately 1.6 million deaths annually (www.who.int/ith/diseases/pneumococcal/en/). These infections are caused by Streptococcus pneumonia, making it an important public health issue (www.who.int/immunization/topics/pneumococcal_disease/en/). *S.pneumoniae* (aka pneumococcus) normally reside in the nasopharynx and upper respiratory tract (www.who.int/ith/diseases/pneumococcal/en/). It causes invasive infections such as meningitis, bacteremia, or bacteremic pneumonia (www.who.int/ith/diseases/pneumococcal/en/). *S.pneumoniae* can also cause non-invasive infections such as non-bacteremic pneumonia, otitis media and sinusitis (www.who.int/immunization/topics/pneumococcal_disease/en/). In North America the incidence of invasive pneumococcal disease (IPD) is 15-49 per 100,000 persons, slightly higher than the European incidence of 11-27 per 100,000 persons [1-3]. There are over 29,500 cases of IPD (75% cases are bacteremic pneumonia) and 3,350 IPD-related deaths each year (www.cdc.gov/abcs/reports-findings/survreports/speu15.html). In 2013, two meta-analyses estimated the prevalence of *S. pneumoniae* in community-acquired pneumonia (CAP) to be 19.3% in North America and 27.3% in Europe [4,5]. In North America, the 2015 data from the Center for Disease Control and Prevention (CDC) shows that *S.pneumoniae* was responsible for 900,000 cases of pneumonia and 400,000 pneumonia-related hospitalizations (www.cdc.gov/abcs/reports-findings/survreports/spneu15.html).

Often S.pneumoniae causes infections in those less than 2 years of age, and 65 years of age or above (http://www.who.int/ith/diseases/pneumococcal/en/) [6]. However, individuals 2 to 64 years of age with co-morbid illnesses such as chronic lung disease, chronic liver disease, cardiovascular disease, chronic renal function, diabetes mellitus, and decreased immune function also have an increased risk of developing pneumococcal disease [7-9]. With an aging population and increasing prevalence of chronic illnesses, the disease and economic burden of pneumococcal-related CAP is of concern and focus of prevention for public health.

In adults 65 years of age and over and individuals between the ages of 19-64 with compromised immunity, two vaccines are available for protection against pneumococcal infections: the 23-valent pneumococcal polysaccharide vaccine (PPV23) and the 13-valent pneumococcal conjugate vaccine (PCV13) (www.cdc.gov/pneumococcal/vaccination.html). The PPV23, contains 23 pneumococcal serotypes: 1, 2, 3, 4, 5, 6B, 7F, 8, 9N, 9V, 10A, 11A, 12F, 14, 15B, 17F, 18C, 19F, 19A, 20, 22F, 23F, and 33F, and has been available since the late 1970s (www.fda.gov/downloads/BiologicsBloodVaccines/Vaccines/ApprovedProducts/UCM218554.pdf.

PPV23 induces antibodies by a T-cell independent mechanism, resulting in a short lived and non- anamnestic response [10]. There is considerable heterogeneity in data reported by studies evaluating efficacy and effectiveness of PPV23 depending on the outcome evaluated by the clinical trials [11,12]. Pooled data from one large meta-analysis has shown purified capsular polysaccharide vaccines in immunocompetent adults with strong evidence of effectiveness against IPD [11]. A second meta-analysis showed that PPV23 was not effective against non-invasive pneumonia in the elderly, and those with chronic medical conditions [12].

By contrast, the 7-valent pneumococcal conjugate vaccine (PCV7), bacterial polysaccharides are covalently conjugated to an immunogenic carrier protein, which induces a T-cell dependent immune response that in turn induces B-cell memory [13]. PCV7 consists of 7 pneumococcal serotypes 4, 6B, 9V, 14, 18C, 19F, and 23F [14]. PCV7 was introduced in the early 2000, as part of routine childhood immunization program [13, 14]. Shortly after, numerous studies showed that PCV7 was not only effective for the prevention of invasive disease, but also in preventing pneumococcal pneumonia, and otitis media in children [13-16]. A decrease in pneumococcal infections in adults has also been seen through herd immunity [16-19]. Most immunization programs have now replaced PCV7 with PCV13, which has 6 additional serotypes - 1, 3, 5, 6A, 7F and 19A [20].

The Advisory Committee on Immunization Practices (ACIP) in the United States[21], the National Advisory Committee on Immunization (NACI) in Canada[22] and the New Zealand Ministry of Health[23] recently recommended PCV13 for healthy adults ≥ 65 years of age with or without chronic illnesses. By contrast, the Australian Technical Advisory Group on Immunization (ATAGI) [24], the Joint Committee on Vaccination and Immunization (JCVI) in the UK (www.gov.uk/government/publications/jcvi-interim-statement-on-adult-pneumococcal-vaccination) and the Standing Committee on Vaccination (STIKO) in Germany [25] continue their recommendation for the use of PPV23 among adults aged ≥ 65 years (60 for Germany), with or without chronic diseases due to limited PCV13 effectiveness evidence. This narrative review will summarize the current evidence regarding the immunogenicity, efficacy, safety and effectiveness of PCV13 vaccine in the prevention of pneumococcal infections in adults.

**Data Sources and Selection:** A MEDLINE literature search from January 1946 to December 2017. Additional references were identified from a review of citations. All English-language randomized trials, observational studies and meta-analyses assessing the immunogenicity, efficacy, effectiveness and safety of PCV13 in adults were evaluated. Clinical guidelines were obtained from their respective government websites.

**Data reporting and interpretation:** Opsonophagocyctic assay (OPA) geometric mean titer (GMT) ratio between vaccines arms were reported as statistically higher when the 95% confidence interval did not include 1. Immunoglobulins geometric mean concentrations (GMC) ratio between vaccines arms were reported as statistically higher when the 95% confidence interval did not include 1. P values reported when available.

## Immunogenicity and Safety

### Immunocompetent adults

Immunogenicity and safety of PCV13 among adults aged 18 to 103 years, with or without prior PPV23 vaccination, has been established through 18 studies [26-44]. Six of the 18 studies were randomized clinical trials comparing PCV13 to either PPV23 [26,27,29-31] or placebo [32] with four also evaluating sequential dosing [27-30] in 6468 adults aged 50 and older (Table 1). Four of the six studies [26,29,31,32] were conducted in pneumococcal vaccination naïve subjects, while two studies [27,30] had subjects with prior PPV23 vaccination experience. In most studies, all immunocompetent subjects were enrolled as long as chronic medical conditions (e.g., cardiovascular, pulmonary, liver diseases including alcoholic liver disease and alcoholism, renal and urinary disorders or diabetes mellitus) had been stable for at least 12 weeks prior to the study period [26-44]. While most studies have reported serotype specific to the OPA GMTs, some have reported anti-pneumococcal immunoglobulin G (IgG) geometric mean concentrations (GMCs) associated with protection efficacy amongst adults for pneumococcal disease [45,46]. The OPA GMTs, and IgG GMCs were obtained at 1 month, 2 months, 1 year and/or 5 years following vaccination, which reflects the functional immune response over time.

**Table 1:** Studies evaluating immunogenicity of 13-valent conjugate pneumococcal vaccine (PCV13) in adults.

Study Name	Design	PPV23 Naïve	Age group	Duration of follow up	PCV13 (N)^1^	PPV23 (N)^1^	PLB (N)	Immunogenicity^2^
Jackson 2013a [26]	R, DB	Y	50-64 years	4 years	Baseline for 60-64 yrs: N=417 (411) At 1 year for 50-59 yrs: N=406	Baseline for 60-64 yrs: N=414 (407)	-	In 60-64 yrs: PCV13 arm had a statistically significantly higher OPA titers in 9/13 serotypes (1,4,6A,6B,7F,9V,18C,19A,23F) compared to PPV23. In 50-59 yrs: PCV13 arm had a statistically significantly higher OPA titers in 9/13 serotypes (1,4,5,6A,6B,7F,9V,14,19A) compared to G2.
Jackson 2013b [27]	R, DB	N	≥70 years	1 year	431	448	-	PCV13 arm had a statistically significantly higher OPA titers in 11/13 serotypes (1,4,5,6A,6B,7F,9V,18C,19A,19F,23F) compared to PPV23.
Greenberg 2014 [29]	R, DB	Y	60-64 years	1 year	482	238	-	PCV13 arm had a statistically significantly higher OPA titers in 11/13 serotypes (1,4,5,6A,6B,7F,9V,18C,19A,19F,23F) compared to PPV23.
Juergens 2014 [30]	R	N	≥65 years	2 years	Baseline for PCV13 with AlPO_4_: N=309 (307) Baseline for PCV13 without AlPO_4_: N=305 (302)	Baseline: 301 (300)		PCV13 (with AlPO_4_) arm had a statistically significantly higher OPA titers and IgG GMCs in 11/13 serotypes (1,3,4,5,6A,6B,9V,18C, 19A,19F,23F) compared to PPV23. PCV13 (without AlPO_4_): had a statistically significantly higher IgG GMCs in 1/13 serotypes (7F) compared to PCV13 (with ALPO_4_).
Shiramoto 2015 [31]	R, DB	Y	≥65 years	4 months	382	382	-	PCV13 arm had a statistically significantly higher OPA titers in 13/13 serotypes.
van Deursen 2017 [32]	R, DB	Y	≥65 years	2 years	1006	-	1005	PCV13 arm had a statistically significantly higher OPA titers in 13/13 serotypes. At 12 months, PCV13 arm had a statistically significantly reached GMFR (OPA titers) in 9/13 serotypes (4,6A,6B, 7F, 14, 18C, 19A,19F, 23F). At 24 months, PCV13 arm had a statistically significantly reached GMFR (OPA titers) in 6/13 serotypes (4,6A,6B, 18C, 19A, 23F).

Abbreviations: PCV13 - 13 valent conjugate pneumococcal vaccine; PPV23 - 23 valent pneumococcal polysaccharide vaccine; PLB- Placebo

1 N is for all randomized subjects and those included in the immunogenicity analyses are included in brackets if they differed from original.

2 All OPA GMT ratios are reported at 1 month after vaccination

In the six studies evaluating PCV13 against PPV23 [26,27,29-31] or placebo [32], subjects in the PCV13 arm had significantly higher OPA GMTRs, for at least 9 of the 13 serotypes (range 9/13 to 13/13 serotypes), one month after vaccination (Table 1). Two studies evaluated OPA GMTs at least 1 year post-vaccination [26,32]. Jackson et al. found OPA titers declined from 1 month to 1 year following PCV13 and PPV23 vaccine administration, although both remained above baseline, and were similar at 1 year follow up [26]. van Deursen et al. reported immunogenicity details up to 24-months and showed that at 1 month, the PCV13 arm had significantly higher OPA GMTRs across all 13 serotypes when compared to placebo [32]. Although, the titers slowly declined with time, they remained above both baseline and placebo at 12 and 24 months post-vaccination, signaling a slow waning of immunity [32].

**Table 2:** Studies evaluating immunogenicity of 13-valent conjugate pneumococcal vaccine (PCV13) in adults in sequential dosing.

Study Name	Age group	Sequential Dosing Interval	PCV13/PCV13 (N)^1^	PCV13/PPV23 (N)^1^	PCV13PPV23 PCV13 (N)^1^	PPV23/PCV13 (N)^1^	Immunogenicity
Jackson 2013b [27]	≥70 years	1 year	At 1 yr: N=391 (372)	-	-	At 1 yr: N=404 (373)	1. PCV13/PCV13 had statistically significantly higher OPA titers in 3/13 serotypes (6A, 6B, 23F) compared to PCV13 alone. 2. PCV13/PCV13 had statistically significantly higher OPA titers in 10/13 serotypes (1,4,5,6A,6B,9V,18C,19A,19F,23F) compared to PPV23 alone. 3. PCV13/PCV13 had statistically significantly higher OPA titers in 12/13 serotypes (1,3,4,5,6A, 6B,7F,9V,19A, 19F, 23F) compared to sequential dose of PPV23/PCV13. 4. PPV23/PCV13 OPA titers did not differ statistically compared to PCV13 alone.
Jackson 2013c [28]	50-64 years	4 years	For 60-64 yrs: N=108 For 50-59 yrs: N=214 (211)	For 60-64 yrs: N=108			For 60-64-year age group: 1. PCV13/PPV23 had statistically significantly higher OPA titers in 10/13 serotypes (1,3,5,6A,6B,7F,18C,19A,23F) compared to PPV23 alone. 2. PCV13/PPV23 had statistically significantly higher OPA titers in 8 serotypes (1,3,5,9V,18C,19A,19F) compared to PCV13 alone. 3. PCV13/PCV13 had statistically significantly higher OPA titers in 7/13 serotypes (1,4,6A,6B,7F,9V,18C,19A,23F) compared to PCV13 alone. For 50-59-year-old age group: 1. PCV13/PCV13 had statistically significantly higher OPA titers in 6/13 (1,4,6A,6B,7F,9V,18C,19A,23F) compared to PCV13 alone. 2. PCV13/PCV13 had statistically significantly higher OPA titers in 5/13 serotypes (4,6A,7F,9V,19A) compared to PCV13/PCV13 in 60-64-year age group.
Greenberg 2014 [29]	60-64 years	1 year	160 (133)	267 (237)	-	223 (199)	1. PCV13/PPV23 had a statistically significantly higher OPA titers in 7/13 serotypes (3,5,6A, 6B,7F,19F,23F) compared to PPV23 alone. 2. PCV13/PPV23 had a statistically significantly higher OPA titers in 11/13 serotypes (1,3,4,5,6B,7F,9V, 18C,19A,19F,23F) compared to PPV23/PCV13. 3. PCV13/PCV13 had a statistically significantly higher OPA titers in 6/13 serotypes (1,3,6A, 6B,7F,9V) compared to PPV23/PCV13. 4. PCV13/PCV13 had a statistically significantly higher OPA titers in 1/13 serotypes (23F) compared to PCV13 alone. 5. PCV13/PPV23 had a statistically significantly higher OPA titers in 1/13 serotypes (3) compared to PCV13 alone. 6. PPV23/PCV13 OPA titers did not differ statistically compared to PCV13 alone.
Juergens 2014 [30]	≥65 years	1 year	At 1 yr: N=136	At 1 yr: N=131	At 2 yr: N=104	-	1. PCV13/PPV23 had a statistically significantly higher OPA titers in 8/13 serotypes (1,3,5,6A, 6B,9V,19F,23F) compared to PPV23 alone. 2. PCV13/PPV23/PCV13 had a statistically significantly higher OPA titers in 3/13 serotypes (6A, 6B,23F) compared to PCV13/PPV23. 3. PCV13/PCV13 had a statistically significantly higher OPA titers in 1/13 serotypes (23F) compared to PCV13 alone. 4. PCV13/PPV23/PCV13 OPA titers did not differ statistically compared to PCV13 alone.

Abbreviations: PCV13 - 13 valent conjugate pneumococcal vaccine; PPV23 - 23 valent pneumococcal polysaccharide vaccine; PLB- Placebo

1 N is for all randomized subjects and those included in the immunogenicity analyses are included in brackets if they differed from original.

2 All OPA GMT ratios are reported at 1 month after vaccination

Jackson et al. conducted a randomized, double-blind study in 1224 immunocompetent, healthy, pneumococcal vaccination naïve adults [26]. They report both local reactions (PCV13 82.2% vs PPV23 75.9%; p=0.052) and systemic adverse events (PCV13 82.6% vs PPV23 82.1%; p=0.907) were comparable between PCV13 and PPV23 in pneumococcal vaccination naïve adults [26]. Individuals aged 50-59 years who received a PCV13 dose experienced higher frequency of both local, (89.6%) and systemic reactions (84.4%) compared to those aged 60-64 years administered either PCV13 or PPV23 [26]. In contrast, Jackson et al. evaluated safety in adults aged 70 years and older with previous PPV23 vaccination at least 5 years earlier, and reported more local reactions (PPV23 64.1% vs PCV13 56.5%; p=0.033) and systemic adverse events (PPV23 68.2% vs PCV13 60.3%;p=0.020) in the PPV23 arm than the PCV13 arm [27]. Prior pneumococcal vaccination had an impact on the frequency of the local and systemic reaction in the subsequent dosing at 1 year interval, both local and systemic reactions were significantly higher in PCV13/PPV23 (86.8%, 78.8%) arm compared to sequential dosing of PCV13/PCV13 (78.5%,70.9%) and PPV23/PCV13 (71.3%, 62.7%) where p<0.001 [29]. Another Jackson et al. study corroborated this finding, who report a sequential dose of PPV23 or PCV13 even after a four-year interval causes higher local and systemic reactions [28]. Frequency of local reactions were highest in PPV23/PPV23 arm (88.7%) followed by PCV13/PPV23 (86.7%) and PCV13/ PCV13 (81.1%). Frequency of systemic reactions were comparable in PPV23/PCV13 (86.4%) and PCV13/PPV23 (87.5%) arms, but lower in PCV13/PCV13 arm (79.1%). No vaccine related, serious adverse events were reported with any dose.

Vaccine sequence assessments of PCV13 and PPV23 were conducted by four studies. As shown in Table 2, among three of those studies the repeat dose was at 1-year[27,29,30], while one study used a dosing interval of 4-years [28]. Three studies demonstrated the initial PCV13 dose enhanced responses to a subsequent PPV23 dose, compared with PPV23 alone [28-30]. Four studies compared the sequential dose of PCV13/PCV13 dose to the initial dose of PCV13/placebo [27-30]. Although the second dose of PCV13 administered a year later did not enhance the first dose response [27,29,30], but a significantly higher response in 7 of 13 serotypes was reported when re-vaccinating after 4 years [28]. Sequential dosing of PCV13/PPV23 elicits statistically higher OPA titers compared to PPV23/PCV13[29] or PPV23/PPV23 [28]. Lastly, another dose of PCV13 after sequential dosing of PCV13/PPV23 was unlikely to enhance the immune response in the study conducted by Juergens et al. where the sequential dose of PCV13/PPV23/PCV13 was comparable to PCV13 only [30].

## Impact of concomitant vaccination on Immuno-genicity

Concomitant administration with influenza vaccines has been evaluated in three studies [33-35]. In a phase 3 randomized and double-blind study, anti-trivalent influenza vaccine (TIV) and anti-pneumococcal immune responses to TIV and PCV13 were evaluated among those 65 years and over received PCV13 concomitant with TIV (Group 1, N=580) followed by placebo after 1 month, concomitant placebo and TIV (Group 2, N=580) followed by PCV13 after 1 month [33]. Non-inferiority was met for all three influenza vaccine antigens as the proportion of participants that achieved ≥4-fold increase in HAI assay for the three influenza viruses were similar in both groups (A/H1N1, 80.3% and 78.6%, respectively; A/H3N2, 58.0% and 62.6%, respectively; and B, 52.2% and 54.0%, respectively). In both groups, 12/13 serotypes (1,4,5,6A,6B,7F,9V,14,18C,19A,19F and 23F) met the predefined IgG GMC ratio non-inferiority criterion of 2-3-fold rise in GMCs. Of interest, IgG GMC ratio of group 1 was statistically significantly lower for 6 serotypes (4, 5, 6A, 14, 19A, and 19F) compared to group 2. Local reactions associated with the concomitant administration of PCV13 and TIV were comparable to those associated with PCV13 alone, but more systemic adverse events were seen with concomitant administration (60.1%) than PCV13 (48.5%), or TIV alone (50.5%); no vaccine-related serious adverse events occurred. The authors concluded that concomitant administration of PCV13 and TIV demonstrated acceptable immunogenicity and safety compared to either vaccine administered alone.

In a second randomized, double-blind, parallel group, phase 3 trial, Frenck et al. evaluated the anti-TIV immune response in 50-59 year old who received TIV followed by placebo (Group 1, N=554) compared to the concomitant PCV13 and TIV (Group 2, N=562) [34]. Additionally, the study evaluated whether the immune responses to PCV13 in group 1 were non-inferior to those in group 2. The proportions of participants that achieved ≥4-fold increase in hemagglutination inhibition (HAI) assay for the three influenza viruses were similar in both groups (A/H1N1, 84.0% and 81.2%, respectively; A/H3N2, 71.1% and 69.5%, respectively; and B, 60.6% and 60.3%, respectively); non-inferiority criterion was met for all three influenza vaccine antigens. In contrast, when evaluating the effect of concurrent administration on PCV13 serotypes, the investigators saw that in group 1, although all serotypes met the predefined IgG GMC ratio non-inferiority criterion relative to group 2, the GMCs were lower in group 1 than group 2. When comparing group 1 with group 2, 5 serotypes did not meet the OPA GMT ratio non-inferiority criterion, and OPA GMTs were significantly lower for 10 serotypes. The authors concluded that co-administration of PCV13 and TIV was well tolerated but associated with lower PCV13 antibody responses. However, given the positive immunologic attributes of PCV13, concomitant administration with TIV should not be contraindicated, but rather dictated by clinical circumstances.

The third study was open-labeled and used adjuvant influenza vaccine (MF59-adjuvant trivalent influenza vaccine (Fluad, Novartis Vaccines and Diagnostics, S.R.L., Siena, Italy) instead of TIV [35]. A total of 1149 subjects aged ≥60 years were randomized in a 1:1:1 ratio in MF59-adjuvant influenza vaccine (MF59-aTIV) + PCV13 (Group 1, N=373), PCV13 alone (Group 2, N=394), or MF59-aTIV alone (Group 3, N=382) arm. Serologic responses for influenza vaccination were assessed using criteria set by the Committee for Medicinal Products for Human Use (CHMP). At least one of the following three criteria were required for each influenza virus strain: (1) GMT-fold increase >2.0; (2) seroprotection rate >60%; or (3) seroconversion rate >30%. Both groups (Groups 1 and 3) met CHMP immunogenicity criteria at one-month post-vaccination. For the influenza A/H1N1 virus, the seroprotection (HI titer ≥40) rate was 88.5% for Group 1 (MF59-aTIV + PCV13) and 91.6% for Group 3 (MF59-aTIV alone) (p=0.18). For the A/H3N2 virus, rates were 98.9% and 99.2% for Groups 1 and 3, respectively (p=0.72). For the influenza B virus, seroprotection rates were 72.7% and 72.3% for Groups 1 and 3, respectively (p=0.92). Overall, seroconversion rates were higher in Group 1 compared to Group 3, and GMTs were more than two-fold higher at one-month post-vaccination for all 3 subtypes irrespective of concomitant administration. For the PCV13 serotypes, the non-inferiority criterion of OPA GMT ratios was met for all 13 pneumococcal serotypes after concomitant administration versus PCV13 alone (Group 1 versus Group 2), but it should be noted that serotypes 1, 3, 5, 19A tended to be higher in Group 2 compared to Group 1. The majority of local reactions were mild in severity and pain at the injection site was more commonly reported by participants who received both vaccines (55.2%) than by those who received PCV13, (41.4%) or aTIV only (29.8%) (p < 0.001). There was no statistically significant difference in systemic adverse events among the three groups except for muscle ache, which was more common in the combination and PCV13 arms. The authors concluded that adjuvant influenza vaccine could be safely administered together with PCV13, although immunogenicity of PCV13 for the four serotypes (1, 3, 5 and 19A, p<0.05) was slightly reduced in the concomitant vaccination group compared to those with PCV13 alone. Results did not show significant immune interference.

### Immunocompromised adults

Use of PCV13 in immunocompromised individuals has been assessed in subjects with Human Immunodeficiency Virus (HIV) [36-38], chronic renal failure [39], and recipients of Hematopoietic Stem Cell Transplant (HSCT) [40]. Lombardi et al. evaluated immunogenicity in HIV-infected pneumococcal vaccine-naïve subjects aged 18-65 years with CD4 counts >200 cells/μL over time [36]. Subjects were recruited into two arms, the first receiving two doses of PCV13 eight weeks apart (N=50) and the second group receiving one dose of PPV23 (N=50). Both vaccines elicited an immune response after the first dose, leading to markedly higher IgG GMCs to each of the antigens compared to baseline values at 48 weeks. Overall, the proportion of subjects achieving seroprotection and seroconversion was comparable between groups and a four-fold rise in IgG GMCs was observed for PCV13/PCV13 in 7/13 serotypes (6A, 6B, 14, 18C, 19A, 19F, 23F) and among PPV23/placebo recipients in 5/13 serotypes (1, 14, 18C, 19A, 19F) at 8 weeks post vaccination. Both PCV13/PCV13 and PPV23/placebo arms sustained a comparable immune response at 24 and 48 weeks. The authors concluded that, in their population of immunologically stable HIV subjects, both vaccines showed comparable immunogenicity and that two doses of PCV13 were as safe and well-tolerated as the single dose of PPV23.

In another study evaluating PCV13 in HIV positive individuals, Bhorat et al. evaluated the immunogenicity of 279 subjects with CD4 counts >200 cells/μL and viral load less than 50,000 copies/mL [37]. Subjects were administered three doses of PCV13, followed by a dose of PPV23 with 1-month intervals between each dose. Statistically significant increases in IgG GMCs and OPA GMTs were observed for all 13 serotypes after one dose of PCV13 compared to baseline, while the increase after doses 2 and 3 were modest. Pain at the injection site was the most common local reaction reported, while muscle pain, fatigue and headache were commonly reported systemic events. The percent of individuals experiencing side effects was the highest after the first dose of PCV13, and decreased with subsequent doses.

In a third study of PPV23-experienced HIV positve individuals, aged ≥18 years with CD4 counts >200 cells/μL and viral load less than 50,000 copies/mL, Glesby et al. evaluated immunogenicity in 329 subjects [38]. Subjects were administered three doses of PCV13 at 6-month intervals and statistically significant increases in IgG GMCs and OPA GMTs were observed for all serotypes one month after the first dose of PCV13, when compared to baseline. Like Bhorat et al., the GMTs at 1 month, after doses 2 and 3, were slightly higher than the levels after dose 1. GMTs in subjects with 1 previous dose of PPSV23 were similar to those with ≥2 previous doses. Local reactions were common with 80% of subjects reporting pain at the injection site after the first dose. The percentage of subjects experiencing pain increased with subsequent vaccine doses. Systemic side effects were also common and seen in 88% of subjects after dose 1 of the vaccine. The most frequent systemic events were fatigue, headache and new generalized muscle pain. Both the systemic and local adverse events were slightly higher in subjects with ≥2 previous doses of PPV23 than in those with 1 previous dose.

Those with renal failure are at increased risk of pneumococcal infections and mortality due to disorders of adaptive immune response [47,48]. Mitra et al. evaluated immunogenicity in 25 End Stage Renal Disease patients on dialysis aged ≥ 50 years who had previously received 1 or more doses of PPV23 [39]. Patients were administered one dose of PCV13, and their IgG GMCs were recorded at 2 and 12 months after dosing. While statistically significant increases in IgG GMCs were observed for all serotypes at two months, significant increases in IgG GMCs remained only for serotypes 5, 19F, 6B, and 18C at 12 months. The GMCs, at 12 months post-vaccination, declined by 38% to 72% compared to those measured at 2 months post-vaccination. The overall rate of response to each individual vaccine serotype varied between 23.5% and 94.1% at 2 months post-vaccination and 23.5% and 65% at 12 months post-vaccination. Pain at the injection site was the most common local reaction. PCV13 induced antibody responses to vaccine serotypes in subjects with ESRD and those on dialysis at 2 months following vaccination. However immune response had declined by 12 months following vaccination.

As *S.pneumoniae* infections are common in recipients of hematopoietic stem cell transplant subjects and vaccination is an important method of prevention, Cordonnier et al. evaluated the immunogenicity of PCV13 in 155 adults and 54 children who had received a HSCT [40]. Subjects were administered four doses of PCV13 (first three doses at one-month intervals and the fourth dose at 6-month interval), followed by PPV23 after a month. Among adult subjects, a four-fold rise increase in IgG GMCs and OPA GMTs was reported for all three doses compared to baseline in 11/13 serotypes (1, 3, 4, 6A,6B, 7F, 9V,14, 18C, 19F and 23F). After the fourth dose of PCV13 and PPV23, a four-fold increase in IgG was noted in 13/13 serotypes compared to baseline. Local pain at injection site and systemic side effects of fever, fatigue and muscle aches were the most common reactions, occurring more frequently after dose 3.

## Efficacy and Safety

To date, there is only one randomized, double-blind, placebo-controlled clinical trial looking at the outcomes of prevention of vaccine-type invasive and noninvasive community-acquired pneumonia in adults 65 years of age or older [49]. The Community-Acquired Pneumonia Immunization Trial in Adults (CAPITA) enrolled 84,496 persons (42,240 received PCV13 and 42,256 received placebo) with a mean age of approximately 73 years (62 to 101) and a mean of 4 years of follow-up. In the per-protocol analysis of first episodes of infections due to vaccine-type strains, CAP occurred in 49 subjects in the PCV13 group and 90 subjects in the placebo group (vaccine efficacy 45.6%; 95%CI 21.8-62.5). Non-bacteremic and noninvasive CAP occurred in 33 subjects in the PCV13 group and 60 subjects in the placebo group (vaccine efficacy 45.0%; 95.2% CI, 14.2-65.3), and invasive pneumococcal disease occurred in 7 persons in the PCV13 group and 28 persons in the placebo group (vaccine efficacy 75.0%; 95% CI, 41.4 to 90.8). Although vaccine efficacy was slightly lower in the modified intention to treat analyses, VT-CAP, non-bacteremic and noninvasive CAP, and invasive pneumococcal disease were all statistically significant (p<0.05). Efficacy persisted throughout the trial (mean follow-up, 3.97 years). The number of serious adverse events (PCV13 0.8% vs Placebo 0.7%; p=0.61) and deaths (PCV13 7.1% vs Placebo 7.1%; p=0.98) were similar across groups, but there were more local reactions in the PCV13 group (PCV13 18.7% vs Placebo 14.3%; p=0.01). Post hoc analyses showed that the vaccine effect began shortly after vaccination and was sustained throughout the duration of the trial (mean follow-up, approximately 4 years) without evidence of waning. In this trial, 30.4% of the study population also received the influenza vaccine at the same time. However, authors have not highlighted any differences in IPD, pneumonia or mortality rates due to concomitant influenza vaccination. The authors identified two limitations of the trial. First, the study was conducted in only the Netherlands, with a homogenous population and a low incidence of IPD, which makes it difficult to generalize to other populations with diverse population and high incidence of IPD. Second, their use of serotype specific urinary antigens with higher sensitivity to detect pneumococcal infection could have overestimated the proportion of vaccine type serotypes, thus resulting in lower efficacy against pneumococcal pneumonia.

**Table 3:** Guidelines for use of the 13-valent conjugate pneumococcal vaccine (PCV13) in adults.

Comorbidities Type	USA [21,50]	Canada [22,79]	United Kingdom [51]	Germany [52]	Australia [24]	New Zealand [23]
**No underlying comorbidities^1^**	PCV13 given at ≥65 years; PPV23 after at least 12 months	PCV13 given at ≥65 years; PPV23 after at least 8 weeks	-	-	-	PCV13 given at age ≥65 years; PPV23 after at least 8 weeks
**Immunocompromising condition^2^ or anatomical/functional asplenia^3^**	PCV13 given at 19-64 years; PPV23 after ≥8 weeks; again after ≥5 years; then at age ≥65 years	PCV13 given at 19-64 years; PPV23 after ≥8 weeks; again after ≥5 years; then at age ≥65 years	PCV13 given at 19-64 years; PPV23 after ≥8 weeks; then at age ≥65 years	PCV13 given at 19-59 years; PPV23 after 6-12 months; again after ≥6 years; then at age ≥60 years	PCV13 given at 19-64 years; PPV23 after ≥8 weeks; again after ≥5 years	PCV13 given at ≥18 years; PPV23 after ≥8 weeks; again after ≥5 years; then at age ≥65 years
**Cerebrospinal fluid leak or cochlear implant**	PCV13 given at 19-64 years; PPV23 after ≥8 weeks^4^	-	PCV13 given at 19-64 years; PPV23 after ≥8 weeks; again after ≥5 years	PCV13 given at 19-59 years; PPV23 after 6-12 months; again after ≥6 years	-	PCV13 given at ≥18 years; PPV23 after ≥8 weeks; again after ≥5 years; then at age ≥65 years
**Hematopoietic stem cell transplant (HSCT)**	-	3 doses of PCV13 starting 3-9 months after transplant (at least 4 weeks apart); PPV23 given 12-18 months after transplant (6-12 months after last PCV13 dose); PPV23 booster 1 year after last dose	PCV13 given 9-12 months after transplant; PPV23 given 12-18 months after transplant (6-12 months after last PCV13 dose)	-	3 doses of PCV13 starting 6 months after transplant (at least 8 weeks apart); PPV23 given 12 months from last PCV13 dose; No more than 3 PPV23 lifetime doses	PCV13 given after transplant; PPV23 given after at least 8 weeks from PCV13; then re-vaccination after 5 years; last dose at age ≥65 years
**Chronic illness or lifestyle risk factors^5^**	PCV13 at age ≥ 65 years, then PPSV23 after at least 12 months	-	-	-	-	PCV13 given at ≥18 years; PPV23 after ≥8 weeks; again after ≥5 years; then at age ≥65 years

Abbreviations: PCV13 - 13 valent conjugate pneumococcal vaccine; PPV23 - 23 valent pneumococcal polysaccharide vaccine;

1 This recommendation is for pneumococcal naïve persons

2 Defined as congenital or acquired immunodeficiency, HIV infection, chronic renal failure, nephrotic syndrome, leukaemia, lymphoma, Hodgkin disease, generalized malignancy, multiple myeloma, solid organ transplant and iatrogenic immunosuppression

3 Defined as sickle cell disease and other haemoglobinopathies, congenital or acquired asplenia, splenic dysfunction and splenectomy

4 Revaccination with PPV23 not required at 5-year mark

5 Defined as chronic heart, lung disease, liver disease or diabetes

6 Defined as smoker, homeless or persons with alcoholism or illicit drug use

All participants who received the study vaccine were included in the safety analysis. Participants used electronic diaries to record any local reactions, systemic events, or the receipt of medications for fever or pain for 7 days after vaccination. In addition, nurses and physicians collected information on serious adverse events, newly diagnosed chronic medical conditions (e.g., asthma, emphysema, hypertension, and cardiac failure) and death to discern whether it was attributable to the vaccine. There were significantly more local reactions within 7 days of vaccination, such as redness, swelling, pain and limited arm movement, in the PCV13 group than placebo (38.4% vs 8.4%; p<0.001). However, there was no difference between the groups with respect to any systemic event within 7 days of vaccination, including fever ≥38°C, fatigue, headache, chills, rash, vomiting, decreased appetite, diarrhea, new or aggravated generalized muscle pain, and new or aggravated joint pain, (PCV13 39.5% vs Placebo 34.7%; p=0.04). There were also no significant differences between the two groups in the frequencies of newly diagnosed chronic medical conditions (PCV13 1.7% vs Placebo 1.2%; p=0.46), serious adverse events (PCV13 0.8% vs Placebo 0.7%; p=.61), or deaths (PCV13 7.1% vs Placebo 7.1%; p=0.98) within one month of vaccination.

## Current Clinical Guidelines

**Figure 1.**
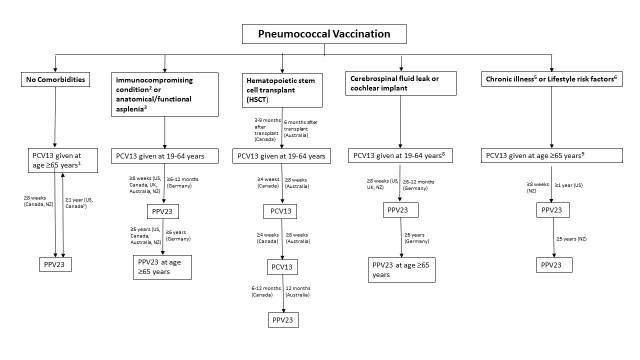
Current Pneumococcal Vaccination Guidelines Abbreviations: PCV13 - 13 valent conjugate pneumococcal vaccine; PPV23 - 23 valent pneumococcal polysaccharide vaccine. ^1^This recommendation is for pneumococcal naïve persons ^2^Defined as congenital or acquired immunodeficiency, HIV infection, chronic renal failure, nephrotic syndrome, leukaemia, lymphoma, Hodgkin disease, generalized malignancy, multiple myeloma, solid organ transplant and iatrogenic immunosuppression ^3^Defined as sickle cell disease and other haemoglobinopathies, congenital or acquired asplenia, splenic dysfunction and splenectomy ^4^Revaccination with PPV23 not required at 5-year mark ^5^Defined as chronic heart, lung disease, liver disease or diabetes ^6^Defined as smoker, homeless or persons with alcoholism or illicit drug use ^7^ACIP (US) and NACI (Canada) recommends those with prior PPV23 vaccination are recommended to wait a year before obtaining PCV13. ^8^Germany’s recommended age is 19-59 years ^9^NZ MoH recommends PCV13 at age ≥18 years and 19-64 respectively

Clinical guidelines on the use of PCV13 in adults are expanding to incorporate new populations, as additional scientific evidence is garnered on the vaccine’s efficacy and safety profile. In addition to the PCV13 dosing scheme, minimum intervals, re-vaccination, additional use of PPV23 and other vaccines vary in different countries (Table 3). By and large all guidelines recommend that PCV13 should be administered before PPV23. Concurrent administration is not recommended, with PPV23 administered at least 8 weeks after PCV13 while some countries suggest a longer period apart (Fig. 1).

As shown in Table 3, the US, Canada and New Zealand (NZ) guidelines recommend routine use of PCV13 for healthy adults aged ≥65 years followed by PPV23 [21-23]. While Canada and NZ recommend waiting at least 8 weeks between PCV13 and PPV23 among pneumococcal naïve adults [22,23]; US recommends waiting at least one-year between PCV13 and PPV23 [50]. At present, no European country recommends the routine use of PCV13 in those ≥65 years. Of note, most guidelines recommend PCV13 after a 12 month wait period in those who have previously received the PPV23 vaccine [22,50].

Australia, Germany, NZ and UK support the routine use of PCV13 for adults aged ≥19 years with immunocompromising conditions (e.g., chronic renal failure, nephrotic syndrome, HIV and leukemia), cerebrospinal fluid leak, functional/anatomic asplenia or cochlear implants [21-24,50-52], followed by administration of PPV23 after ≥8weeks and at ≥5 years, then at the age of 65 years. Germany recommends an interval of 6-12 months between PCV13 and PPV23 with a re-vaccination gap of at least 6 years. For hematopoietic stem cell transplant recipients, Canadian and Australian guidelines suggest 3 doses of PCV13 after the transplant, followed by PPV23 [22,24]. However the UK and New Zealand guidelines recommend one dose of PCV13 given 9-12 months after transplant, followed by PPV23 [23,51].

For patients who have chronic medical conditions (such as chronic heart disease, lung disease, liver disease or diabetes), smoke or are alcoholic, ACIP recommends PCV13 should only be administered after adults reach 65 years of age, followed by PPV23 after a year [50]. None of the other countries recommend PCV13, except New Zealand which recommends PCV13 for those aged ≥18 years followed by PPV23 after at least 8 weeks [23].

## Discussion

Previously, much of the burden of disease associated with *S. pneumoniae* was in children less than 5 years of age and adults over the age of 65 years. After the introduction of the conjugate pneumococcal vaccine as part of routine childhood immunization programs, many studies reported a significant reduction in incidence of invasive pneumococcal disease among children, particularly the serotypes present in the vaccines, with reductions of 80% for vaccine-type IPD and approximately 60% for overall IPD rates [53]. Modest benefits have also been observed in non-invasive infections such as acute otitis media, CAP and subsequent hospitalizations [53-59]. As transmission of *S. pneumoniae* occurs in large part from children to adults, an important consequence of the decrease in circulating vaccine serotypes has been the significant degree of herd immunity protection provided to adults [60,61]. With studies reporting >90% decline in pneumococcal disease due to vaccine serotypes in older children and adults [62], as well as a 56% reduction in the incidence of vaccine-type community acquired pneumonia [63,64].

Despite the significant protection confered to adults by the childhood immunization program, it is evident that herd immunity alone cannot significantly reduce the burden of pneumococcal infections in the adult population. One of the main problems is serotype replacement, whereby the incidence of IPD and pneumococcal pneumonia caused by non-vaccine serotypes increases [65-68]. In a recent meta-analysis that examined data from 38 studies (14 countries) where PCV7 was administered, serotype 19A (which is not in the 7-valent vaccine) was the most predominant cause of childhood IPD, accounting for 21.8% (95%CI 18.6±25.6) of cases. In countries that introduced higher valent PCVs (19A is represented in these vaccines), the overall contribution of 19A was lower at 14.2% (95% CI 11.1±18.3) and non-PCV13 serotypes contributed to 42.2% of childhood IPD cases, with predominant non-PCV13 serotypes being 22F, 12F, 33F, 24F, 15C, 15B, 23B, 10A, and 38 (descending order) [67].

Another reason for evaluating the direct use of PCV13 in adults, instead of relying on herd immunity from children, is an increase in the incidence of CAP, hospitalizations, and mortality due to cardiovascular and lung-related complications with increasing age [69-72]. This phenomenon is thought to be related to immunosenescence, defined as changes in immune function associated with natural ageing due to decreases in the number and function of antigen-presenting cells as well as B and T cells [73]. Jackson et al. reported a dramatic surge in the estimated incidence of CAP with age, from 18.2 per 1000 person-years in individuals aged 65-69 years to 52.3 per 1000 person-years among those aged over 85 years [69]. Pneumonia-related hospitalizations, complications and death is also more common as individuals age, illustrated by Jain et al. who enrolled 2488 patients in an active surveillance study between 2010 and 2012 [74]. The annual incidence of pneumonia was 24.8 cases (95% CI, 23.5 to 26.1) per 10,000 adults, with the highest rates among adults 65 to 79 years of age (63.0 cases per 10,000 adults) and those 80 years of age or older (164.3 cases per 10,000 adults). In a large Spanish cohort study, the mortality rates for those 65 years of age and over with CAP was 10.3% compared to 2.2% in patients under 65 years [71]. Among patients aged 75 and above, with severe CAP requiring mechanical ventilation, mortality rates were as high as 53% [72].

For decades, the PPV23 vaccine has been recommended in adults 65 years and older and in individuals with chronic diseases and/or immunosuppression, to protect adults against CAP. However, considering IPD and pneumococcal-related CAP rates continue to be high in the elderly, the effectiveness of PPV23 is doubtful. The meta-analysis conducted by Moberley et al. included 25 studies (18 RCTs involving 64,852 participants and seven non-RCTs involving 62,294 participants). Their analysis of the RCTs found strong evidence of PPV23 efficacy against IPD (OR 0.26, 95%CI 0.14 to 0.45) [11]. However, efficacy against all-cause pneumonia in high-income countries in either the general population, (OR 0.71, 95% CI 0.45 to 1.12) or in adults with chronic illness (OR 0.93, 95% CI 0.73 to 1.19) was not significant, nor was it associated with substantial reductions in all-cause mortality (OR 0.90, 95% CI 0.74 to 1.09) [11]. A second meta-analysis by Huss et al. included 22 trials involving 101 507 participants [12]. Although the initial analysis showed that PPV23 was effective for presumptive pneumococcal pneumonia (N=11 trials, RR 0.64, 95% CI 0.43-0.96) and all-cause pneumonia (N=19 trials, RR 0.73, 95% CI 0.56-0.94), when the authors repeated their analysis after excluding poor quality trials, the protective effect of PPV23 was eliminated [12]. In addition, little benefit was seen with the vaccine for the elderly and those with chronic illness [12]. In large part, the contradictory results are due to the different inclusion criteria of the two meta-analyses that resulted in a number of different studies being included [11,12]. For example, the Cochrane review included two large studies that were excluded by Huss et al. because the material examined to diagnose IPD included lung aspirates [75]. For the second study, a number of subjects volunteered to be vaccinated with PPV23 rather than undergoing randomization [76]. While meta-analyses results are contradictory, recent population based studies have shown PPV23 to be effective against pneumococcal pneumonia and vaccine serotype IPD compared to those without pneumococcal vaccination [77,78].

For these reasons, the focus has shifted to evaluating the use of the conjugate pneumococcal vaccines in adults. Immunogenicity data evaluating PCV13 suggest that it has better immune response to the vaccine serotypes compared to the polysaccharide vaccine [26,27,29-31] and that the response is sustained over time [27]. These studies demonstrate that PCV13 is well tolerated, safe among adults, and causes minor side effects such as injection site pain, redness and swelling, or mild systemic reactions such as fever, body pains, vomiting or decreased appetite. Individuals who are PPV23 naïve and receive PCV13 as an adult experience more local as well as systemic side effects [26,29,31,32], with older adults having less adverse events compared to the younger adults [26]. Studies involving sequential dosing show that adults receiving PPV23 followed by another dose of PPV23 have the highest adverse event profile [28] and those receiving concomitant influenza vaccine with PCV13 reported higher rates of local and systemic reactions compared to PCV13 alone [35].

CAPiTA, a large randomized clinical trial, suggests excellent vaccine efficacy and safety data in immunocompetent adults within the general population. This trial excluded subjects with immunocompromised conditions, and those living in nursing home and long-term care facility, consequently PCV13 vaccine efficacy is unknown in these populations. PCV13 showed superiority to placebo in the prevention of vaccine-type CAP, the primary endpoint; but also, against the two secondary endpoints - reducing the number of episodes of confirmed vaccine-type nonbacteraemic and noninvasive CAP (vaccine efficacy 45%) and confirmed vaccine-type IPD (vaccine efficacy 75%). The number of adverse events were higher in the vaccine arm compared to placebo, but only as it related to minor local events.

Despite the availability of the CAPiTA trial for PCV13, there is a plethora of unanswered questions in the absence of head-to-head RCTs comparing clinical efficacy and effectiveness of PCV13 to PPV23. Nevertheless, the clinical guidelines now recommend administration of PCV13 for those 65 years of age and above and younger adults aged 18 years of age or over with chronic medical conditions and/or immune-compromised in few countries. The comparative arm for CAPiTA was placebo and not PPV23, which is the current standard for adult vaccination in high-income countries, so it does not address the question of whether PCV13 is suitable replacement of PPV23. Additionally, when CAPiTA trial began, PCV13 was not recommended for children in the country of study (the Netherlands), so impact of PCV13 serotypes suppression by the herd effect is unknown. In countries with a substantial decrease in vaccine-specific serotypes, it is unclear if there are additional benefits in an adult vaccination program, alongside the with routine childhood immunization. However, as stated in the clinical guidelines section, some countries recommend PCV13 for all adults 65 years of age and above, with ongoing surveillance to determine its impact on IPD, nonbacteremic pneumonia and hospitalization rates.

## Conclusion

The burden of invasive pneumococcal disease among adults has declined in countries where the conjugate pneumococcal vaccine is part of the childhood immunization program, due to the increasing herd immunity. However, pneumococcal pneumonia in older adults remains significant. It is unlikely that the use of the polysaccharide vaccine alone is sufficient to reduce the pneumococcal disease incidence, hospitalizations and/or mortality associated with community-acquired pneumonia. While PCV13 has a well-established immunogenicity and safety profile in adults, there is sparse data on sequential or multiple dosing, efficacy and effectiveness amongst adults. Therefore, only a few countries, including US and Canada, have adopted PCV13 along with PPV23 for routine adult immunization. Other countries, like Germany and the UK, need additional effectiveness data before the adoption of PCV13 in adult immunization programs.
